# The prevalence of ataxia telangiectasia mutated (ATM) variants in patients with breast cancer patients: a systematic review and meta-analysis

**DOI:** 10.1186/s12935-021-02172-8

**Published:** 2021-09-08

**Authors:** Masoumeh Moslemi, Maryam Vafaei, Pouria Khani, Marzieh Soheili, Reza Nedaeinia, Mostafa Manian, Yousef Moradi, Ehsan Sohrabi

**Affiliations:** 1 Department of Medical Genetics and Molecular Biology, Faculty of Medicine, Iran University of Medical Sciences Tehran (IUMS), Tehran, Iran; 2 Department of Genetics, Faculty of Medicine, Shahid Sadoughi University of Medical Sciences, Yazd, Tehran, Iran; 3 Department of Medical Genetics, Faculty of Medicine, Tehran University of Medical Sciences, Tehran, Iran; 4 Faculty of Medicine, Kermanshah University of Medical Sciences, Kermanshah, Iran; 5 Pediatric Inherited Diseases Research Center, Research Institute for Primordial Prevention of Non-Communicable Disease, Isfahan University of Medical Sciences, Isfahan, Iran; 6 Department of Medical Laboratory Science, Faculty of Medicine, Kermanshah Branch, Islamic Azad University, Kermanshah, Iran; 7 Department of Immunology, School of Medicine, Iran University of Medical Sciences, Tehran, Iran; 8 Social Determinants of Health Research Center, Research Institute for Health Development, Kurdistan University of Medical Sciences, Sanandaj, Iran; 9 Department of Epidemiology and Biostatistics, Faculty of Medicine, Kurdistan University of Medical Sciences, Sanandaj, Iran

**Keywords:** ATM variants, Breast cancer, Prevalence, BRCA, Meta-analysis

## Abstract

Breast cancer is the most common cancer in women, and its high mortality has become one of the biggest health problems globally. Several studies have reported an association between breast cancer and ATM gene variants. This study aimed to demonstrate and analyze the relationship between ATM gene polymorphisms and breast cancer prevalence rate. A systematic literature review was undertaken using the following databases: Medline (PubMed), Web of sciences, Scopus, EMBASE, Cochrane, Ovid, and CINHAL to retrieve all cross-sectional studies between January 1990 and January 2020, which had reported the frequency of ATM variants in patients with breast cancer. A random-effects model was applied to calculate the pooled prevalence with a 95% confidence interval. The pooled prevalence of ATM variants in patients with breast cancer was 7% (95% CI: 5−8%). Also, the pooled estimate based on type of variants was 6% (95% CI: 4−8%; I square: 94%; P: 0.00) for total variants¸ 0% (95% CI: 0−1%; I square: 0%; P: 0.59) for deletion variants, 12% (95% CI: 7−18%; I square: 99%; P: 0.00) for substitution variants, and 2% (95% CI: 4−9%; I square: 67%; P: 0.08) for insertion variants. This meta-analysis showed that there is a significant relationship between ATM variants in breast cancer patients. Further studies are required to determine which of the variants of the ATM gene are associated with BRCA mutations.

## Introduction

Breast cancer is a global health problem and has the highest worldwide incidence among women. Approximately 1.7 million patients have diagnosed with breast cancer annually [1]. Furthermore, breast cancer is a significant cause of cancer-related death in women [2, 3]. The high rate of cancer progression and metastasis are two unsolved problems associated with this high mortality rate [4]. Breast cancer is a complex disorder; its etiology has not been entirely explained. Like other cancers, genetic factors have an essential role in the familial and sporadic forms of breast cancer [4]. Twin studies indicate that the heritability of breast cancer is between 27 and 31% [5, 6]. Three genetic factors are involved in breast cancer progression: high penetrance genetic mutations such as those in the *BRCA1* and *BRCA2* genes [7–9], intermediate penetrance variants such as those in *ATM*, *BARD1*, *PALB2*, and *CHECK2* genes [10], and low-penetrance variants such as SNPs. The first and second mutation groups explain approximately 22% of breast cancer risk. Still, it seems that a complex interaction between low penetrance susceptibility genes and environmental factors is vital for the development and progression of breast cancer [10–13].

DNA repair systems protect the genome against mutagens and have an essential role in cell cycle regulation. Different gene mutations in these systems can increase the risk of cancer development [11, 14]. Ataxia telangiectasia mutated (ATM) is a protein kinase enzyme with a crucial role in the DNA repair system, especially in DNA double-strand repair. This gene is located on chromosome 11q 22–23 and includes 66 exons [15]. ATM acts as an intracellular sensor that becomes active in response to DNA double-strand break and then phosphorylates many downstream tumor suppressor genes such as BRCA1, p53, chk2, and chk1 [16]. Thus, it is a strong candidate for mutation in cancer multistage development [17]. ATM biallelic mutation causes an autosomal recessive disease known as Ataxia-Telangiectasia [18]. Ataxia Telangiectasia was described in 1926 and is characterized by telangiectasia, cerebellar Ataxia, neurological abnormalities, immunological deficiency, hypersensitivity to ionizing radiation (IR), and predisposition to cancer [19]. Truncated mutations in the ATM gene inhibit its expression and cause Ataxia Telangiectasia; however, missense mutations change its function and are common in cancers [20, 21]. It was reported that ATM gene expression decreased in breast cancer tissues and cells compared to controls [22]. Various studies show that classic A-T heterozygote women have a 4-fold increased risk for breast cancer [23–25]. Some studies have reported an association of ATM gene mutations with breast cancer, and some have evaluated the prevalence of this gene variant in breast cancer. Still,the exact prevalence of ATM mutations in breast cancer is unclear, and there is no systematic review on the prevalence of ATM variants in breast cancer. We aimed to undertake a systematic review and meta-analysis of the prevalence of ATM variants in breast cancer from different countries, the association of these variants with the BRCA status of patients, and the prevalence of other variants in the ATM gene.

## Materials and methods

All approaches used in this study were in agreement with the Preferred Reporting Items for Systematic Reviews and Meta-Analyses statement (PRISMA), and the protocol had been registered in the International Prospective Register of Systematic Reviews (PROSPERO), under the registration number of CRD42018114400.

### Search terms and complex search syntax

**Fig. 1:**
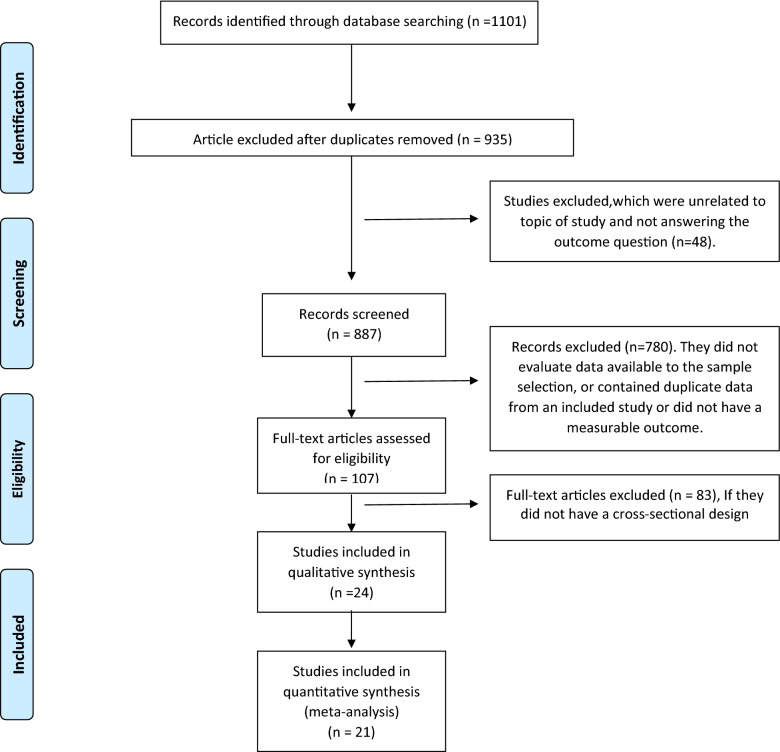
Preferred reporting items for systematic reviews and meta-analyses diagram with search strategy and screening results

Medline (PubMed), Web of sciences, Scopus, EMBASE, Cochrane, Ovid, and CINHAL databases were searched to evaluate the prevalence of ataxia telangiectasia mutated (ATM) variants in breast cancer. In current study “breast carcinoma”, “breast tumor”, “breast neoplasm”, “breast neoplasms”, “breast cancer”, " breast cancers”, “breast tumors”, “mammary cancer”, “mammary cancers”, “breast carcinomas”, “mammary carcinoma”, " mammary carcinomas”, “ataxia telangiectasia mutated”, “ataxia telangiectasia mutated proteins”, “Ataxia Telangiectasia Mutated Proteins”, “ATM”, " mutation”, “mutations”, “variant” and “variants” keywords were searched in all mentioned databases. All reference lists of primary articles were manually reevaluated by two individuals (MM, ES) separately to avoid missing any papers. First, the authors reviewed the titles and abstracts to select the appropriate articles. The results of the primary search were reviewed, and some articles were eliminated in this step. After reviewing the entire text of the selected articles, the inclusion and exclusion criteria were set by two researchers separately (MM and ES) (Fig. 1).

### Eligibility criteria

Articles were included in the current study using the following criteria: (1) evaluation of the epidemiological aspects of ATM variants in patients with breast cancer; (2) only full-text articles; (3) Cross-sectional studies. Case reports, reviews, animal studies, and cohort studies were excluded. The authors resolved all disputes during the data collection, compilation, and data analysis. The exclusion criteria for this study were unrelated studies, duplicate data, and the studies that have not answered the outcome questions, have not assessed the available data, and have not had a cross-sectional design.

### Data extraction

The first ‘author’s name, publication date, sample size, country, type of variant, prevalence, BRCA status were extracted from all studies and statically analyzed. A data extraction form was created based on our group discussion and piloted according to 10 different studies types, then was modified and used by the data extractor. All processes from systematic search to final data extraction were undertaken independently by two researchers (Kappa statistic for agreement for quality assessment: 0.75). Both authors assessed any controversy,and, in case of dispute, the data was evaluated by the third author (YM).

### Risk of bias

Two of the authors (MM and ES) performed a qualitative evaluation of the studies based on the Newcastle-Ottawa Quality Assessment Scale (NOS) was performed by two of the authors (MM and ES). This scale is designed to evaluate the qualitative evaluation of observational studies. According to NOS, each study was examined by six items in three groups; selection, comparability, and exposure. Stars were given to each item, and the maximum score is 9. The external discussion method was used in case of differences in the score given to the published reports. Finally, the papers were categorized as low, moderate, and high risk. The Strengthening the Reporting of Observational Studies in Epidemiology (STROBE) checklist was also completed for all articles.

### Statistical analysis

The DerSimonian-Lirad random-effect model was used to pool the prevalence of ATM in patients with breast cancer (and 95% confidence interval estimation) using the Metaprop command in Stata 14. Cochran Q and I2 tests were used to investigate the heterogeneity and variance between studies. The Q statistic tells us whether there is statistically significant heterogeneity among the studies or not. I2 determines the amount of heterogeneity quantitatively. The range of I2 is from 0 to 100. In this study, we categorized it into three levels; low (25%), moderate (50%), and high (75%). The funnel diagram, ' ‘Egger’s teste, and graphs were used to evaluate publication bias. In the Egger regression model, the ratio of the effect size on the standard error, which is the standard index (z-score), is taken as the dependent variable and predicts its value over the standard error inverse (1/SE). Subgroup analyses were performed to examine any confounding factors that may influence the prevalence of the disease. Subgroup analyses were performed for BRCA status, countries, and variants of ATM. We used P-value to decide on the statistical hypothesis test results. All two-way statistical tests were considered as α = 0.05.

## Results

### Study characteristics

The searches retrieved a total of 1101 original-research articles, the titles of which were examined by two independent reviewers. Forty-eight articles were excluded because they were not related to the topic of study. Eight hundred and sixty-three studies were excluded because of the unavailability of the full text. After all, they did not evaluate the data available for the sample selection because they contained duplicate data from an included research or did not have a cross-sectional design (Fig. 1). Finally, the remaining 24 articles were retained for analysis (Table 1).

**Table 1:** The characteristics of studies examining the prevalence of ATM variants in women with breast cancer

Authors	Years	Sample size	Country	Type	Prevalence %	BRCA status
de Souza Timoteo et al. [26]	2018	157	Brazil	All	10.53	Not determined
Prodosmo et al. [27]	2016	496	Italy	All	1.41	Negative
Broeks et al. [28]	2000	82	Amsterdam	All	8.54	Negative
				Substitution	3.66	Negative
Broeks et al. [29]	2008	443	Netherlands	Substitution	51	Positive
				Deletion	0.41	Positive
				Insertion	0.53	Positive
				All	24.15	Positive
Szabo et al. [30]	2004	961	France	Substitution	0.83	Negative
Atencio et al. [31]	2001	45	New York	All	6.67	Not determined
Aloraifi et al. [32]	2015	104	Ireland	All	4.81	Negative
Fostira et al. [33]	2018	102	Greece	All	1.96	Positive
Birrell et al. [34]	2005	32	Australia	Substitution	25	Not determined
Lindeman et al. [35]	2004	495	Australia	Substitution	1.41	Negative
Brunet et al. [36]	2008	43	Spain	Deletion	2.33	Negative
				All	30.23	Negative
				Substitution	6.98	Negative
				Insertion	6.98	Negative
Soukupova et al. [37]	2008	161	Czech Republic	All	1.86	Negative
				Substitution	0.62	Negative
Olsen JH et al. [24]	2001	1090	Denmark	All	0.46	Negative
La Paglia et al. [38]	2009	122	France	All	2.46	Negative
				Substitution	6.56	Negative
Minion et al. [39]	2015	353	USA	All	0.85	Negative
Pinto et al. [40]	2016	94	Portugal	All	5.13	Positive
Buys et al. [41]	2017	658	Utah	All	9.73	Negative
Ohnami et al. [42]	2017	60	japan	All	3.33	Positive
Bozhanov et al. [43]	2010	145	Bulgaria	All	7.59	Positive
Dörk et al. [44]	2001	192	Germany	Deletion	0.52	Not determined
				All	2.08	Not determined
		1000		Substitution	23.5	Not determined
Buchholz et al. [45]	2004	91	Texas	All	38.46	Not determined
		286		Substitution	11.19	Not determined

## Quantities results

**Fig. 2:**
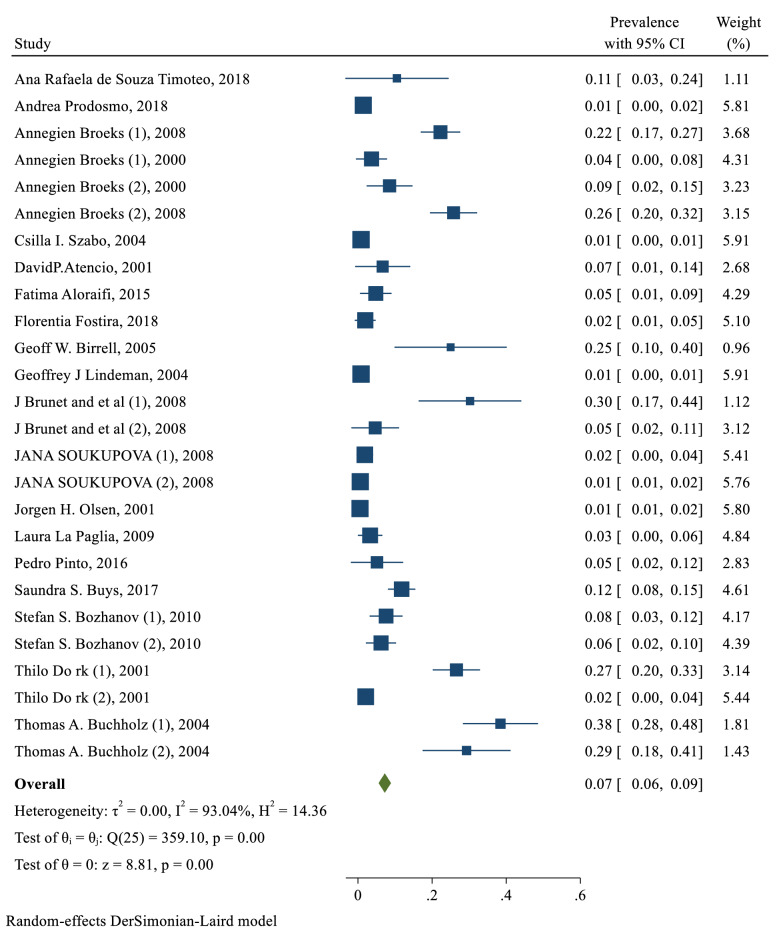
The pooled prevalence of ATM in patients with breast cancer

The overall pooled prevalence of ATM in patients with breast cancer was 7% (95% CI: 6−9%; I square: 93%; P: 0.00) (Fig. 2).

### The prevalence of different ATM variants in studies

We classify ATM variants into four groups: All variants, deletion, insertion and substitution variants. The effect range of this study was between 0.05 and 0.08. The prevalence of substitution variants was higher than other variants. Deletion and insertion were seen rarely in Breast cancer according to this study. The pooled estimate based on type of variants was 0% (95% CI: 0−1%; I square: 0%; P: 0.59), 2% (95% CI: 4−9%; I square: 67%; P: 0.08), 12% (95% CI: 7−18%; I square: 99%; P: 0.00) and 6% (95% CI: 4−8%; I square: 94%; P: 0.00) for deletion, insertion, substitution variants and total, respectively (Table 2).

**Table 2:** The subgroup analysis of the prevalence of ATM variants in patients with breast cancerbased on ATM variants, BRCA status, and continents

Variables	Subgroups	Pooled prevalence (% 95 CI)	Heterogeneity assessment		
			I_square_ (%)	Q	P _Heterogeneity_
ATM variant	All	6% (4–8%)	94	289	0.00
	Deletion	0% (0–1%)	0	1	0.59
	Insertion	2% (4–9%)	67	3	0.08
	Substitution	12% (7–18%)	99	743	0.00
BRCA status	Positive	11% (7–15%)	99	604	0.00
	Negative	3% (2–4%)	85	107	0.00
	Not determined	12% (6–18%)	98	336	0.00
Continents (population)	American	9% (4–14%)	96	125	0.00
	Asian	5% (1–11%)	80	10	0.01
	European	7% (5–8%)	98	939	0.00

### The prevalence of ATM in patients with breast cancer in the world by BRCA status

The studies were divided into positive, negative, and not determined groups based on BRCA mutations. Positive groups had BRACA1/2 mutations, and negative groups do not. Some studies did not determine the status of BRCA mutations, and we classify these data as an undetermined group. The pooled prevalence of ATM in patients with breast cancer in several BRCA status was 7% (95% CI: 5−8%; I square: 97%; P: 0.00). The pooled estimate based on type of variants was 3% (95% CI: 2−4%; I square: 85%; P: 0.00), 11% (95% CI: 7−15%; I square: 99%; P: 0.00) and 12% (95% CI: 6−18%; I square: 98%; P: 0.00) for negative, positive, and not determined, respectively (Table 2). It appears that the prevalence of ATM variants in the BRCA positive group was more than negative.

### The prevalence of ATM in patients with breast cancer in the world by countries

The pooled prevalence of ATM in patients with breast cancer in several continent was 7% (95% CI: 5−8%; I square: 98%; P: 0.00), 5% (95% CI: 1−11%; I square: 80%; P: 0.01) and 9% (95% CI: 4−14%; I square: 96%; P: 0.00) for European, Asian and American population, respectively (Table 2).

### Publication bias

**Fig. 3:**
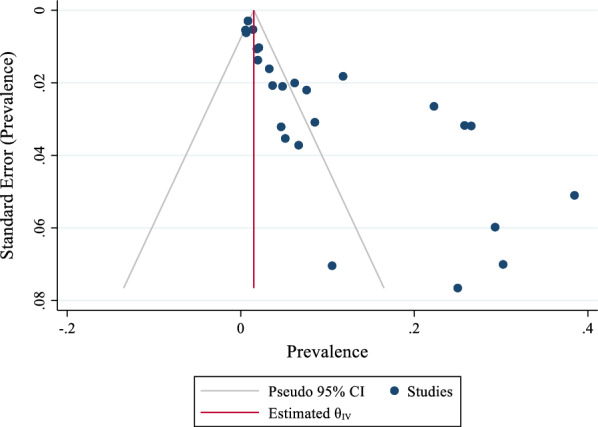
The funnel plot of pooled prevalence of ATM in patients with breast cancer

The results of ‘Egger’s test showed no publication bias in the pooled prevalence of ATM in patients with breast cancer (coefficient = 0.098, P = 0.98). Also, the funnel plot is reported in Fig. 3.

### Meta-regression

Meta-regression results on the heterogeneity of studies showed that the sample size and mean age of study participants had no significant effect on the prevalence of ATM in patients with breast cancer.

## Discussion

ATM is an essential protein that protects the genome from the effects of genotoxic agents, such as ionizing radiation. This protein can detect DNA double-strand breaks and directly or indirectly activate many other proteins that are important in the DNA repair system [47]. According to these studies, there is an association between ATM variants and breast cancer risk. In the current study, the prevalence of ATM variants in breast cancer patients was evaluated. Our results showed significant differences among countries. ATM variants were highest in breast cancer patients from the USA,t but these variants were not involved in the prevalence of patients from northern Europe (Finland, Denmark, and Sweden.)

In 1996, the association of Ataxia–telangiectasia gene (ATM) mutation heterozygosity with breast cancer risk was reported from New York. This study reported that 6.6% of breast cancer patients in the USA had this ATM variant [46]. So, it can be concluded that there is a high prevalence of ATM variants in the United States. This issue is consistent with our results. Different studies on the ATM variants and the prevalence of breast cancer have been undertaken in northern Europe. Most studies reported that there was no association between common variants in the ATM gene and breast cancer susceptibility in patients from Sweden, Finland, and Denmark [47, 48]. Therefore, these studies are consistent with our findings.

There are several variants of the ATM gene reported in different populations [49]. In our study, ATM variants were classified into three groups: deletion variants, insertion variants, and substitution variants. Deletions and insertions were reported in few cases. The incidence of substitution variants was more common than other variants. Most of the studies indicated the prevalence of the A-T substitution variant in the ATM gene [50, 51]. Another study reported that regardless of the common A-T mutation, substitution and especially missense mutations are the most common ATM variants in breast cancer patients.

Furthermore, a frameshift deletion in exon 28 of the ATM gene was reported in this article in one breast cancer patient (among 192 patients) that produce a truncated protein [52]. Another study reported one deletion 1563delAG and one insertion 2572insT in ATM gene among 190 breast cancer patients [53]. It seems that deletion and insertion in ATM genes are infrequent in breast cancer patients. There is little literature about the association of these variants,with breast cancer patients. There is little literature about the association of these variants withbreast cancer disease. We classified our data into BRCA positive and BRCA negative groups for identifying the association of ATM variants with BRCA1/2 mutation in breast cancer patients. The results showed that ATM variants in the BRCA positive group are more common. In some studies, the ATM mutation (causing Ataxia Telangiectasia) was reported as a risk factor for breast cancer patients who did not have a BRCA1/2 mutation. These studies suggested that ATM is as crucialas BRCA2 in breast cancer [54, 55]. Another study indicathat ATM mutations and BRCA1 mutations are associated with breast cancer patients [56].

Our results may support this latter study and suggest that there is a acumulative effect of ATM and BRCA1 mutations that increase the risk of breast cancer incidence. Overall it seems that the eprevalence of ATM variants ismore common in the USA than in other countries. Among different variants, isubstitutions are the most common, and there is an association between ATM variants and BRCA mutations in breast cancer incidence.

## Data Availability

Not applicable.

## Abbreviations

ATM: Ataxia telangiectasia mutated
IR: Radiation
PRISMA: Reviews and meta-analyses statement
PROSPERO: Prospective register of systematic reviews
ATM: Ataxia telangiectasia mutated
